# Cross talk of the first-line defense TLRs with PI3K/Akt pathway, in preconditioning therapeutic approach

**DOI:** 10.1186/s40591-015-0041-7

**Published:** 2015-05-30

**Authors:** Fatemeh Pourrajab, Mohammad Baghi Yazdi, Mojtaba Babaei Zarch, Mohammadali Babaei Zarch, Seyedhossein Hekmatimoghaddam

**Affiliations:** School of Medicine, Shahid Sadoughi University of Medical Sciences, Professor Hessabi 11 BLV, Shohadaye Gomnam BLV, Yazd, Iran P.O. 8915173149,; Department of Clinical Biochemistry and Molecular Biology, School of Medicine, Shahid Sadoughi University of Medical Sciences, Yazd, Iran; School of Paramedicine, Shahid Sadoughi University of Medical Sciences, Yazd, Iran

**Keywords:** TLRs, PI3K/AKT, Signaling, Cross talk

## Abstract

Toll-like receptor family (TLRs), pattern recognition receptors, is expressed not only on immune cells but also on non-immune cells, including cardiomyocytes, fibroblasts, and vascular endothelial cells. One main function of TLRs in the non-immune system is to regulate apoptosis. TLRs are the central mediators in hepatic, pulmonary, brain, and renal ischemic/reperfusion (I/R) injury. Up-regulation of TLRs and their ligation by either exogenous or endogenous danger signals plays critical roles in ischemia/reperfusion–induced tissue damage. Conventional TLR-NF-κB pathways are markedly activated in failing and ischemic myocardium. Recent studies have identified a cross talk between TLR activation and the PI3K/Akt pathway. The activation of TLRs is proposed to be the most potent preconditioning method after ischemia, to improve the cell survival via the mechanism involved the PI3K/Akt signaling pathway and to attenuate the subsequent TLR-NF-κB pathway stimulation. Thus, TLRs could be a great target in the new treatment approaches for myocardial I/R injury.

## Review

### Introduction

Toll-like receptors (TLRs), the first line of host defense against microbial infection, play a pivotal role in the induction of both innate and adaptive inflammatory responses. However, recent evidence suggests that TLR-mediated innate and immune responses contribute to organ ischemia/reperfusion (I/R) injury [1]. In hemodynamic stresses and in the response of pressure overloads, TLRs are activated in response to ligands and initiating an immune response [1–4].

TLRs are the evolutionarily conserved transmembrane receptors that recognize conserved microbial motifs called pathogen associated molecule patterns (PAMPs). PAMPs include bacterial lipopolysaccharide (LPS, recognized by TLR4), lipoteichoic acid (recognized by TLR2), unmethylated CpG-DNA (recognized by TLR9), and single or double stranded RNA (recognized by TLR3) [2–5]. TLRs also recognize endogenous ligands called damage-associated molecule patterns (DAMPs), which are released from body cells under pathological conditions [1–4].

DAMPs include heparan sulfate, hyaluronic acid, fibrinogen, high mobility group box 1 (HMGB1), heat shock proteins (HSPs) and oxidized phospholipids [6].

DAMPs interact with TLRs, resulting in activation of MyD88- dependent nuclear factor-κB (NF-κB) signaling pathway. NF-κB is an important transcription factor that regulates numerous gene expression including inflammatory cytokines, such as TNF-α, IL-1ß and IL-6, etc. [7, 8]. TLRs also activate MyD88- independet signaling pathway, resulting in the production of interferons [1, 2, 5].

### TLR ligands induce protection against I/R injury through a preconditioning and/or activation of PI3K/Akt dependent mechanisms

TLRs are the key players in pathogenesis of I/R injuries in heart, brain, liver, renal and rejection of transplants [9, 10]. Activation of TLR-mediated innate immune and inflammatory responses after reperfusion results in a positive feedback loop characterized by an accelerated cytokine and chemokine release and subsequent leukocyte infiltration to the ischemic/reperfused site. The enhanced inflammatory status in the inflamed organ depresses cell function and leads to cell damaged and organ failure [8, 10, 11]. Therefore, TLRs are assumed as potential targets for therapeutic approaches in I/R injuries.

**Fig. 1:**
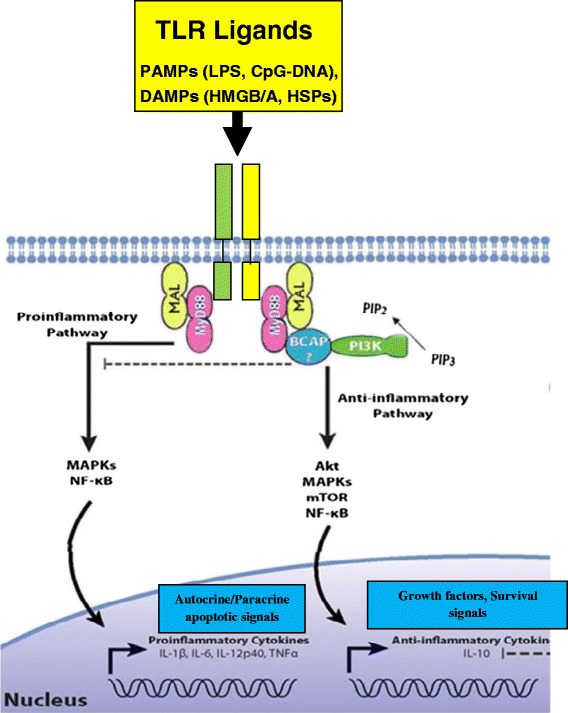
All TLRs signal through the common MyD88-dependent pathway. MyD88 signals via downstream kinases eventually leading to the activation of NF-κB and inflammatory cytokine production. TLR preconditioning triggers cross activation of both MyD88-dependent and Trif-dependent pathways with PI3K/Akt to protect the heart against I/R injury. TLR activation initiates an adaptive immune respons to maintain organ function through both the PI3K survival and NF-κB signaling pathways and moderate expression of proinflammatory cytokines IL-1 ß, IL-6, and TNF-α. Activation of PI3K/Akt signaling limits pro-inflammatory and apoptotic events induced by TLR-NF-κB pathway through an endogenous compensatory mechanism and protects against I/R injuries. But, exaggerated activation of TLRs leads to a positive-feedback-regulation loop in inflammatory pathway and robuset activation of TLR-NF-κB, which subsequently results in cardiac injury and heart impairment

Interestingly, recent studies have shown that stimulation of TLR2/3/9 by their ligands will induce cardiac protection through ischemic or anesthetic preconditioning mechanisms [10–13]. In addition, TLR2, TLR4, and TLR9 ligands have also been reported to induce a protection against ischemic injury through preconditioning mechanisms [7, 14–17]. Through preconditioning mechanism, TLR ligands can activate phosphoinositide 3 kinase (PI3K) signaling [9, 16–18]. PI3Ks and its downstream target serine serin /threonine kinase Akt (PKB), are a conserved family of signal transduction enzymes which constitute an endogenous negative feedback regulator and/or compensatory mechanism, limits pro-inflammatory and apoptotic events in response to injurious stimuli, prevents cardiac myocyte apoptosis and protects myocardium from I/R injuries [17, 19, 20]. Several studies have identified cross talks between TLR signaling and the PI3K/Akt pathway [9, 17–19, 21]. Activation of PI3K/Akt involves the survival pathway of IGF-I signaling and leads to activation of anti-apoptotic and protective genes. In particular, data demonstrate that TLR-induced cardioprotection is mediated through activation of both PI3K/Akt and MEK/ERK dependent mechanisms. Activation of PI3K/Akt signaling has been shown to prevent cardiac myocyte apoptosis and protect the myocardium from I/R injury [11, 13, 17–19]. PI3K/Akt pathway phosphorylates ERK pathway and factors Bim/BCL2. Activation of PI3K/Akt inhibits Bax conformational change, thus preventing Bax translocation and integration into mitochondrial membrane. PI3K/Akt activation also phosphorylates Bim, leading to dissociation of Bim from BCL2. Accordingly, PI3K inhibition abolishes TLR-induced cardioprotection following I/R injury. PI3K/Akt signaling induces an anti-apoptotic function through a mechanism involving Raf/MEK/ERK pathway and Bim/BCL2/Bax factors. Increased level of phospho-ERK involves activation of ERK signaling. ERK can be activated by Ref-mediated MEK signaling. The Raf/MEK/ERK signaling pathway phosphorylates Bad, resulting in its inactivity. This process allows BCL2 to form homodimers and process an anti-apoptotic response. Activation of Raf/MEK/ERK also induces Bim phosphorylation, resulting in Bim disassociation from BCL2. BCL2 then binds to Bax and prevents Bax formation of homodimers and activation. The PI3K/Akt and Raf/MEK/ERK signaling pathways are synergistically regulated by TLR activation and there is a crosstalk between both signaling pathways [11, 13, 17–19, 22]. Accordingly, preconditioning administration of small doses of TLR synthetic ligands, induces a protection against I/R injury in heart and brain [11, 13, 17, 18, 23, 24]. The protection would be through a special anti-apoptotic cross talking mechanism between PI3Ks and NF-κB signaling pathways. Activation of PI3K/Akt-dependent signaling has been shown to limit pro-inflammatory and apoptotic events in response to injurious stimuli by an endogenous compensatory mechanism to protect the myocardium from I/R injury (Fig. 1) [9, 14–17].

Intriguingly, there are several reports of animal models demonstrating that prior administration of sub-lethal doses of TLR ligands protects against subsequent lethal I/R injuries. They confer a “preconditioning-like” mechanism, similar to those observed in ischemic or anesthetic preconditioning [4, 16, 17, 19, 23, 24]. The cardioprotective effects of TLR agonists have been addressed to induction of PI3K/Akt signaling pathway. The studies have clarified a cross talk between TLR and PI3K/Akt signaling pathways [16, 17, 19, 25].

The mentioned studies declare that TLR-cardiac preconditioning would be a well-documented treatment to trigger endogenous survival network to protect the heart against I/R injuries [1, 4, 11, 26, 27].

### TLR4 programed activation as a strategy to trigger the survival pathway in I/R injury

In the solid organ transplantation, TLRs play provital role in promoting graft rejection [1].

Accordingly, systemic and intra-graft inflammatory responses that occur during I/R event, would be markedly reduced in the programmed signaling of TLR4. Programmed activation of TLR4 confers a potent cardiac protection against I/R injury via a MyD88-dependent mechanism [25, 28].

TLR4 signaling on both donor and recipient cell types would mediate the robust early inflammatory responses that occur after I/R condition [19, 29]. The inflammatory reactions occur through reperfusion event whose the outcome is further tissue injury. The degree of tissue injury is especially dependent on the excessive ROS/RNS production during reperfusion, and is dependent on the anoxic phase. The reperfusion phase is well characterized by tissue infiltration of neutrophils and monocytes, cytokine release, complement deposition, endothelial dysfunction, along with activation of platelet and coagulation cascade [19, 30].

In early inflammation of I/R phase after transplantation, contribution of TLR4-putative activator HMGB1 is required, as well as, the co-receptor CD14 and the intracellular adaptor proteins MyD88 and Trif [31–33]. TLR4 become activated in the presence of endogenous molecules DAMPs, in particular HMGB1, released from damaged cells or ischemic/reperfused tissues [1].

The remarkable dependency of TLR4 on HMGB1 in I/R injury of cold organ preservation and transplantation, but not on the other endogenous ligands, such as heparan sulfate or oxidized phospholipids are preferentially considered [34]. After I/R of the grafts, HMGB1 reveals a striking translocation out of the nucleus from damaged cells. In several models of I/R injury, administration of neutralizing antibodies against HMGB1 ameliorate the inflammatory response via systemic reducing of IL-6 and TNFα and ICAM-1 expression [31–33]. Also, a combination treatment with brief doses of anti-inflammatory and antiatherogenic agents Serp-1 and CsA causes indefinite graft survival with normal histology. The treatment has been defined to attenuate TLR2, TLR4 responces through repressing the expression of signaling mediator MyD88 [19]. Notable, in the absence of MyD88, transplantation of skin grafts across minor histocompatibility antigens is possible [35].

In this aspect, the mechanism by which TLRs mediate inflammatory responses after I/R, is mainly depended on MyD88 which is very critical in the pathway. However, both Trif and MyD88 must be absent to permit transplantation across major histocompatibility barriers [1].

All TLRs, except TLR3, signal through the common MyD88-dependent pathway. MyD88 signals via IRAK-1 and other downstream kinases including IKKβ and IκB, eventually leading to the activation of NF-κB and inflammatory cytokine production. TLR3 exclusively, and TLR4 partly signals via Trif-dependent pathway [36].

In the presense of exaggerated activation of TLRs and in a positive feedback loop in the heart for example, the pathway becomes impaired and leads to heart failure [37, 38]. The molecular mechanisms by which TLRs mediate the induction of adaptive heart failure, in response to pressure overload and ventricle wall stresses, is accordingly through the substantially robust activation of NF-κB pathway [12, 39]. While, systemic deficiency of TLR signalling for example, TLR2, TLR4, or intermediate signaling molecule MyD88, leads to attenuated myocardial inflammation, smaller infarction size, and better preserved ventricular function after ischemic injury [40, 41].

However, consistantly several studies have reported that programmed preconditioning with TLR ligands triggers cardiac protection against I/R injuries, in a menner mimicing the ischemic/anesthetic cardiac preconditioning [1, 4, 11].

**Fig. 2:**
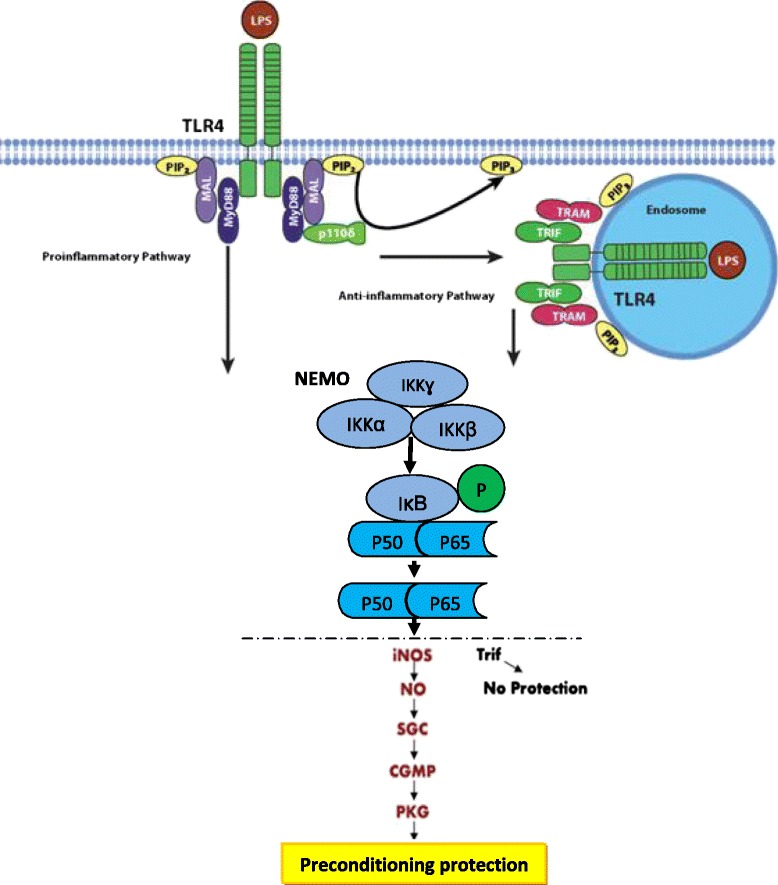
TLR preconditioning triggers cross activation of both MyD88-dependent and Trif-dependent pathways with PI3K/Akt. TLR3 exclusively, and TLR4 partly signals via Trif-dependent pathway. A proposed mechanism for the TLR4-MyD88-dependent mechanism to protect against cardiac I/R. TLR4 activation by its ligand, leads to MyD88-dependent iNOS induction and increased NO production. NO protects myocardium via sGC- and cGMP/PKG-dependent mechanisms. Trif is not required for the TLR4-NO-mediated cardiac protection. LPS, lipopolysaccharide; iNOS, inducible nitric-oxide synthase; MyD88, myeloid differentiation factor 88; sGC, soluble guanylate cyclase; PKG, protein kinase G; TLR, toll-like receptor; Trif, TIR-domain-containing adaptor protein inducing interferon-β–mediated transcription factor

Remarkably, the cardioprotective effect of TLR ligand preconditioning usually occurs between 12 and 24 h after a small dose administration of ligand. According to references, it is assumed that TLR preconditioning triggers cross activation of both MyD88-dependent and Trif-dependent pathways with PI3K/Akt to protect the heart against I/R injury (Figs. 1 and 2), which is abolished by cycloheximide, suggesting a mechanism involving de novo synthesis of cardioprotective proteins [4, 17, 19, 42].

Additinally, the survival pathway PI3K/Akt have in turn a cross talk with both the extracellular signal-regulated kinase 1/2 (ERK1/2) and IκB kinase β (IKKβ) pathways, which is known to be respocible for the LPS-induced TLR4 cardiac protective effects [9].

TLR4 also mediates to induce nitric-oxide synthase (iNOS), and soluble guanylate cyclase (sGC) trough MyD88 and Trif activation pathways. Remarkably, nitric oxide is implicated as key intracellular mediators responsible for both ischemic preconditioning, which promotes cell survival pathway through both cGMP-dependent and cGMP-independent/ caspase-3 nitrosylation mechanisms. The sGC is downstream main effector of nitric oxide and responsible for nitric oxide-elicited biological effects. Nitric oxide binds to the heme moiety of sGC and activate the enzyme to generate cGMP (Fig. 2) [43, 44].

### TLR9 preconditioning reprograms the subsequent responces to TLRs

From the in vivo experiments, it has been discovered that stimulation of TLR9 with its ligands would be the most potent method to attenuate the pro-inflammatory response to the other TLR ligands [45, 46]. Accordingly, TLR9 preconditioning by CpG-DNA (ODN) attenuates ischemic injuries in the brain and liver, through down-regulation of NF-κB pathway and its transcriptional activity [4, 47]. TLR9 agonist ODN attenuates myocardial I/R injury and protects cells against spontaneous apoptosis and prevents trauma-hemorrhage which causes cardiac dysfunction, through activation of PI3K/Akt signaling pathway [17]. The pre-treatment of mice with TLR9 ligand significantly decreases myocardial infarct size and improves cardiac function after I/R events, which indicating TLR9 cardio protective effects against I/R injuries [48]. In I/R and septic mice model, systemic administration of ODN, 72 h prior to brain ischemia reduced ischemic damage up to 60 % [24]. It has been discovered that preconditioning with TLR9 ligand would be a potent tool to reprogram the subsequent TLR2/4/5-stimulation response, in vivo. Despite the well-defined role of TLR2 and TLR4 in protection against I/R injuries, but TLR9 poses a particular role in protecting both heart and brain [18, 23].

The CpG-DNA administration, either as pretreatment or by continuous infusion after the onset of cardiac ischemia, significantly improve LVEF after I/R injury and was observed to attenuate inflammatory cardiac dysfunction, while the same administration induced no detectable change in cardiac physiology in healthy models.

According to the models, TLR9 agonist ODN activates both NF-κB and PI3K/Akt signaling pathway and their transcriptional activity. Activation of NF-κB pathway, however, on its own is able to explosively turn on a broad set of inflammatory cellular programs, because of its powerful nature, but there are a number of negative feedback mechanisms in place to control NF- κB pathway [49].

Herein, TLR moderate activation would inhibit inflammatory pathway and biases signaling toward the PI3K/Akt pathway via inducing the NF-κB pathway inhibitors TNFAIP3, its associated protein TNIP1, NFKBIA, and also an additional inhibitor TRIM30a, which are further up-regulated by activated PI3K/Akt pathway to protects cells against apoptosis [45, 46].

**Fig. 3:**
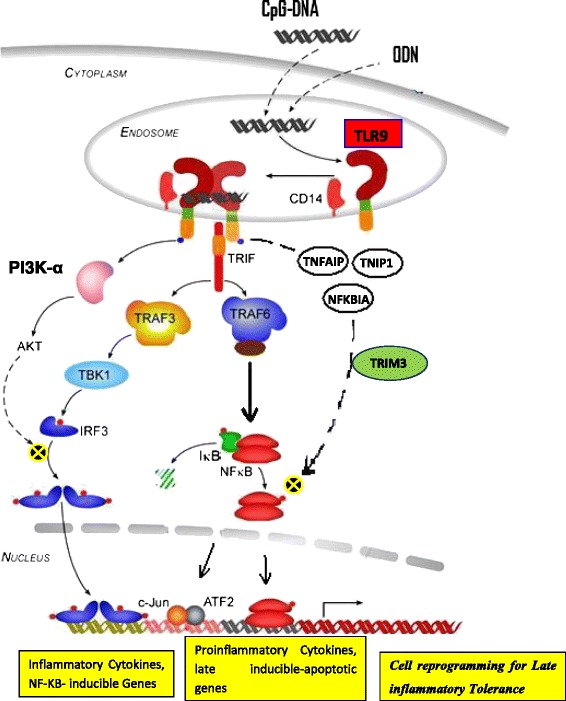
A negative feedback cross talk between PI3Kα/Akt and NF-κB signaling pathway by a local brief stimulation of TLR9, a potent preconditioning tool for programming the subsequent responses to I/R injuries and stimulation by TLR2, TLR4, or TLR5 ligands. TLR9 ligation with CpG-DNA leads to receptor tyrosine phosphorylation and subsequent activation of the p85 subunit of PI3K. The PI3K-α/Akt pathway is subsequently activated in response to TLR9 ligation. Activation of PI3K/Akt pathway protects cells against apoptosis, through up-regulation of the NF-κB pathway inhibitors TNFAIP3/TNIP1/NFKBIA/TRIM30. TLR9 agonist activates both NF-κB and PI3K/Akt signaling pathway. TLR moderate activation directs to PI3K/Akt pathway through up-regulating NF-κB pathway inhibitors TNFAIP3, its associated protein TNIP1, NFKBIA, and also an additional inhibitor TRIM30a, to inhibit inflammatory pathway. Activation of PI3K/Akt signaling protects cells against apoptosis, through further up-regulation of the NF-κB pathway inhibitors TNFAIP3/TNIP1/NFKBIA/TRIM3. TLR9 signaling acts as a dominant activator of the isoform PI3Kα-Akt pathway during I/R injury and in the preconditioning mechanism

The mechanism for down-regulation of NF-κB pathway and its cross talk with PI3K/Akt signaling pathway is according to the several reports and their assumed model for pathway activation (Fig. 3) [4, 45–47].

The PI3K/Akt signaling pathway acts as an endogenous negative feedback regulator and/or compensatory mechanism, to limit pro-inflammatory and apoptotic events in response to injurious stimuli. Recently, a cross talk between TLR signaling and the PI3K-α/Akt pathway have been identified [16, 50, 51].

In the preconditioning mechanism, TLR9 signaling acts as a dominant activator of the isoform PI3Kα-Akt pathway during I/R injury [17, 22].

PI3K and its downstream target Akt are a conserved family of signal transduction enzymes protecting specially myocardium from I/R injuries [52]. According to the references, TLR9-ligation preconditioning would lead to the activation of PI3K-α/Akt pathway, via causing tyrosine phosphorylation of the receptor and subsequent association of the PI3K subunit p85 (Fig. 3) [17–19, 50].

The PI3Kα isoform antagonizes pathological cardiac hyperthrophy and belongs to class I_A_ PI3Ks which are not activated by GPCRs, but are activated by insulin-like growth factor-1 (IGF-1) or other tyrosine kinase receptors/cytokine receptors [53, 54]. Physiological cardiac growth is performed by class I_A_ PI3Ks pathway including PI3Kα, PI3Kβ and PI3Kδ [54]. Remarkable, the transgenic PI3Kα mice are resistant to pathological cardiac hyperthrophy and cardiac dysfunction induced by pressure overload [21]. Apparently, the ODN mechanism for in vivo cardioprotection lies in the up-regulation of PI3K-p110α/p-Akt to protects from heart failure and to prevent cardiac cell death, as reflected in reduced infarct size and in inhibition of cardiac cell death in rats [50, 51].

Whereas, The G protein-coupled receptor (GPCRs)-depended signaling pathway activaties tha PI3Ks (PI3Kγ) that belongs to the class I_B_, a heterodimers of p110γ and an adaptor subunit. The activated class I_B_ PI3Ks are responsible for the prohypertrophic effects of hypertrophic agents noradrenaline, angiotensin II, and endotheilin-1. The Gβγ subunit of G proteins is associated with activated class I_B_ PI3Ks (PI3Kγ) [55]. The activated prohypertrophic PI3Kγ, in turn cross talks with downstream signaling mediators Akt/ERK1/2. Noteworthy, mouse model with genetic knockout of PI3Kγ is resistant to isoproterenol-induced pathological cardiac hyperthrophy and heart dysfunction, accompanied by attenuated activation of Akt and ERK1/2 pathways [55].

Finally, ODN administration also leads to increased levels of phospho-ERK and activation of the Raf/MEK/ERK signaling pathway [56]. Activation of Raf/MEK/ERK pathway causes anti-apoptotic effects through mechanism involving Bim/BCL2/Bax. Both, the Raf/MEK/ERK and PI3K/Akt pathways are synergistically regulated and there is a positive crosstalk between them [57].

Thereby, preconditioning with TLR ligands can emerge an early expression of NF-κB inhibitors which suppress late inflammatory responses and repressing further TLR stimulation [45, 46].

### Release of DAMPs and positiv- feedback-regulation loop of TLR signaling

All known TLRs are expressed in the heart, whose stimulation results in a NF-κB mediated pro-inflammatory response and decreased contractility [58]. In the early phase, these receptors signal predominantly through the ubiquitous transcription factor, NF-κB [59].

After open heart surgery, in chronic pressure overload and in patients with unstable angina or in patients with acute myocardial infarction, DAMPs such as HSP60, HSP70 and HMGB1 are released and signaling through putative receptors TLR2 and TLR4 [60, 61].

In response to chronic stimulation, TLR2 and TLR-4 mediate up-regulation of cardiac TNF-α, IL-1β, and NO and induction of adaptive cardiac hypertrophy [62, 63].

As stress response biomarkers, HMGB1, HSP60 and HSP70 mediates activation of NF-κB and synthesis of pro-inflammatory cytokines IL-1ß and TNF-α [64], in a 2-step mechanism, in the first step response, IL-1ß and TNF-α are induced, required for the second step response in which IL-6 is induced. The first step response contribute to cardiac adaptation to pressure overload, whereas sustained pressure overload and resultant prolonged inflammatory responses induce a shift to the second step response and IL 6–dominant inflammation, which cause adverse cardiac remodeling and heart failure. Because of a positive feedback loop between NF-κB activity and IL-1ß, the over activation of TLR4 or TLR2/NF–κB pathway would lead to cardiac hypertrophy [65].

In acute phase, alarming biomarkers such as HMGB1 are released from damaged cells and also from activated platelets which causes inhibition of mesenchymal stem cells (MSCs) migratory responses via stimulation of TLR2/TLR4 expressed on MSCs. HMGB1 for example activates both TLR2/TLR4 and the transmembrane multi-ligand receptor of immunoglobulin superfamily [66, 67].

Following cardiac injury such as in myocardial infarction, serum level of HMGB1is significantly increased, peaking 12 h after infarction, and is associated with adverse clinical outcomes including pump failure, cardiac rupture, and in-hospital cardiac deaths [61, 68].

HMGB1/TLR4 interaction causes down-regulation of growth factor signaling which interfere with recruitment of MSCs following cardiac injury [61, 68]. Prevention of MSC recruitment to apoptotic site is in part driven by down-regulation of hepatocyte growth factor (HGF) receptor MET on MSCs. HGF is known to be anti-apoptotic [69], pro-angiogenetic [70], and immunosuppressive [71] which confer cardioprotection [72].

Bone marrow MSCs play a critical role in tissue repair and contribute to myocardial recovery [68]. Thereby, blocking TLR4 improves efficacy of MSC-based therapy and their infiltration into the damaged target tissue. It seems a novel mechanism for myocardial repair and regeneration [66].

However, a completely abolished inflammatory response is detrimental for repair events because inflammation is critical for proper wound healing [73, 74]. For example, corticosteroid administration reduces IS but results in aneurysm formation and rupture of the myocardium after infarction. But, programmed activation of TLRs axis induces IGF-1 signaling [8], whereupon contributes to adaptive cardiac function in response to pressure overload, performed by class I_A_ PI3Ks pathway including PI3Kα, PI3Kβ and PI3Kδ [8, 75, 76].

In addition, miR-146 which is up-regulated by TLR4 and pro-inflammatory stimuli IL-1 and TNF-α [77], in a negative-feedback-regulation loop is able to negatively control TLR4 pathway and its downstream cytokine signaling, in human cells. MiR-146 directly targets the NF-κB-dependent genes IRAK1 and TRAF6 (the key kinases downstream of TLR4 signaling) [78, 79]. The impairment of IRAK1/TRAF6 regulation by miR-146 would result in prolonged activation of TLR4 and its downstream signaling, which resulting CAD pathogenesis.

## Conclusions

In organ I/R injuries, in hemodynamic stresses and in response to pressure overloads, conventional TLR-NF-κB pathways are markedly activated. Over-activation of the pathway initiates an immune response which could result in the failing of affected organ. DAMPs signals (HMGB1, HSPs, oPL) released from damaged cells or ischemic/reperfused tissues, interact with TLRs which subsequently activate NF-κB inflammatory pathway. In a manner mimicking the ischemic/anesthetic cardiac preconditioning, all TLRs are able to limit pro-inflammatory and apoptotic events in response to injurious stimuli by a compensatory mechanism which involving PI3K/Akt pathway to protect from I/R injuries. The protection would be depended on the intracellular signaling mediators MyD88 and Trif, through a cross talk mechanism between TLR-NF-κB and PI3K/Akt signaling pathways. The proposed preconditioning mechanism involves the PI3K/Akt signaling pathway to attenuate the subsequent robust stimulation of TLR-NF-κB pathway. In summary, programmed ligation of TLRs would trigger a preconditioning mechanism to protect ischenic heart against I/R injuries .

TLR agonist programmed preconditioning, significantly decreases I/R-induced damages and improves cardiac function following myocardial I/R event. Hence, programmed activity of TLR pathway would hold a great promise in the new therapeutic approach for I/R injuries.
